# Willingness to pay for a quality-adjusted life year for depressive disorders compared to heart disease based on population preferences

**DOI:** 10.1007/s11136-021-02772-x

**Published:** 2021-02-15

**Authors:** Laura Ulbrich, Christoph Kröger

**Affiliations:** 1 Department of Psychology, University of Hildesheim, Hildesheim, Germany; 2 Department of Clinical Psychology and Psychotherapy, University of Hildesheim, Universitätsplatz 1, 31141 Hildesheim, Germany

**Keywords:** Willingness to pay, Quality-adjusted life year, Health utility, Depression, Heart disease

## Abstract

**Purpose:**

According to estimations of the World Health Organization, depressive disorders, and cardiovascular disease will be the leading causes for global burden of disease in 2030. The aim of the present study was to estimate the value a representative sample of the German population places on quality-adjusted life years (QALYs) for depressive disorders compared to heart disease.

**Methods:**

A representative sample of *N* = 967 of the German general public was randomly presented with one of two hypothetical health-loss scenarios: One version of the questionnaire presented respondents with health loss due to depression, while the other version dealt with health loss due to experiencing a heart disease. Respondents were asked to indicate their willingness to pay (WTP) for four hypothetical health-gain scenarios with different treatment options.

**Results:**

In the depression questionnaire median WTP values ranged from 1000 to 1500 EUR; in the heart disease questionnaire from 1000 to 2000 EUR. Results of the Mann–Whitney *U*-Test and Median Test indicate higher WTP values for heart disease compared to depressive disorders when QALY gains were minor and stretched over a long period of time, and when treatment with bypass operation (rather than treatment with ECT) was offered. Zero WTP was significantly higher in all scenarios of the depression questionnaire in comparison to the hearth disease questionnaire.

**Conclusion:**

Results indicate that respondents valued the necessity of paying for treatment higher when presented with heart disease compared to depression.

**Supplementary Information:**

The online version contains supplementary material available at 10.1007/s11136-021-02772-x.

## Introduction

In the healthcare sector, with its limited resource settings, cost-effectiveness analyses are important in terms of input to decision-making; they are used as guidelines in priority setting, resource allocation, and reimbursement decisions. The preferred metric of health benefits in cost-effectiveness analyses and measure of years lived in full health is the measure of quality-adjusted life years (QALYs) [1, 2]. It combines the impact of health changes on both, health-related quality of life and quantity of life years and facilitates the comparison of different interventions within a disease or in comparison with other diseases [3]. Measuring preferences for health improvements, the demand-side value of a person’s willingness to pay (WTP) in gaining a QALY is one relevant component in the interpretation of the results from health economic evaluations [1]. Several studies have tried to estimate the value of a QALY through WTP method (e.g., [3–5]). In a study across nine European countries (‘EuroVaQ study’), a total of *N* = 17,657 respondents was presented with different hypothetical health-gain scenarios and asked to state their WTP. Median WTP per QALY values ranged from $1100 to $2300 [3]. A systematic review including 24 studies on WTP per QALY found that WTP estimates range from €1000 to €4,800,000, with median WTP of €24,226 per QALY. The authors conclude that WTP per QALY seems to be related to several different contextual factors (e.g., size and type of QALY gain valued) and that the assumption that “a QALY is a QALY is a QALY” seems to be untenable [6]. A recent study of WTP per QALY in Japan came to a similar conclusion, that the use of a uniform price threshold may not reflect diverse preferences, which seem to be based on several factors, such as illness type and severity [7].

### Global burden of disease

Estimations of the World Health Organization (WHO) predict unipolar depression to be the leading cause of GBD in 2030, followed by coronary heart disease [8]. A measure quantifying the burden of a disease in terms of mortality and morbidity is the measure of disability-adjusted life years (DALYs). DALYs for a disease or health condition are calculated as the sum of the years of life lost (YLL) due to premature mortality in the population, and the years lost due to disability (YLD) for people living with the health condition or its consequences [9]. The number of all-age YLDs attributed to depressive disorders has increased tremendously over the past decades: depressive disorders are among the three leading causes of YLD [10] and DALYs [11]. Cardiovascular diseases (CVDs), a group of disorders of the heart and blood vessels [12], is estimated to be the leading cause of mortality and morbidity worldwide [13]. In 2016, 17.9 million people died due to cardiovascular diseases, and 85% of these deaths were due to a heart attack [14]. In addition to the person-related burden of a disease, the economic burden can be estimated in terms of direct costs (e.g., costs of hospitalization, psychotherapy, medication) and indirect costs (e.g., reduced productivity and disability insurance; for example, see [15]). For the United States, the total economic burden of cardiovascular diseases is estimated at $320.1 billion [16] and the economic burden of major depressive disorder was estimated at $210.5 billion [17].

To date, no study has attempted to quantitatively assess the degree to which a European population values a QALY gain for a specific physical illness in comparison to a QALY gain for a specific mental disorder, which would allow comparison between both health-gain values. As potential recipients of medical services and payers of social insurance contributions, it would be desirable to know the value a representative population sample places on QALY gains for mental and physical health [4].

### Study aims

The aim of the present study was to investigate the value a representative sample of the German population^1^ places on QALY gains for mental and physical health. Respondents were presented with one of two surveys and were asked to indicate their WTP for different scenarios offering QALY gains regarding a depressive episode or a heart disease. We hypothesize:

1. that WTP per QALY is higher in all presented scenarios of the heart disease questionnaire in comparison to the equivalent scenarios of the depression questionnaire and;
2. that zero WTP is higher in all scenarios of the depression questionnaire compared to the heart disease scenarios.

Additionally, to investigate if the results from the EuroVaQ study are applicable to the presented illness-specific scenarios, we tested if the differences in mean WTP are significant across selected questions for respondents answering each respective pair of questions. The scenario-specific hypotheses are specified after introducing the illness-specific scenarios in “Health gains valued” section.

## Method

### Questionnaire

Question feasibility and validity were examined by pilot respondents (*n* > 10), who were asked to comment on the clarity of the presented scenarios. Their feedback was used to improve the wording of the health state descriptions and the presentation of the questions.

On the first page of the survey, respondents were informed about the objective of the study and had to give consent to start the questionnaire. Respondents were introduced to the hypothetical scenario that no sickness funds exist in Germany and that therefore, they would not have to pay premiums or contributions for health insurance. Instead, they would have to pay for every medical service out of their own pocket and had saved money for such medical expenditures.

**Fig. 1:**
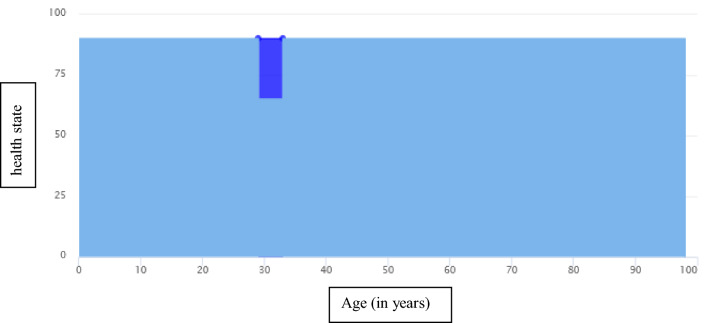
Example of a health gain scenario (Scenario A). The scenario is tailored to respondent’s age, health state and life expectancy. This figure shows a 28-year-old with a life expectancy of 98 years (on the *x*-axis) and a current health state of 90 points (out of 100; on the *y*-axis). The respondent is presented with a loss in quality of life of minus 25 points in one year’s time for a period of 4 years. If the respondent would be willing to pay for treatment his/her health could be restored (dark blue) and s/he would live his/her life at the current health state until his/her expected death

The concept of measuring health on a visual analog scale was introduced, and generic health-state descriptions were used to indicate different levels of health on the scale. These consisted of three European Quality of Life 5 Dimensions 3 Level Version (EQ-5D-3L [18]) health states, and numerical valuations derived from survey values [19] to ensure comparability to the EuroVaQ questionnaire. The respondents were then asked to answer various demographical questions (e.g., age, income, health insurance), to estimate their life expectancy, and to rate their current health on a visual analogue scale (European Quality of Life Visual Analogue Scale (EQ-VAS [18])) with values between 0 and 100. The Patient Health Questionnaire (PHQ-2 [20]) and EQ-5D-3L [18] were used to briefly assess symptoms of depression and current health-related quality of life. The respondents were then randomly presented with a description of the impact of one of two diseases: heart attack or depression. The description included typical symptoms and their impact on everyday life, as well as mortality rates. The detailed translations of the health state descriptions are displayed in Online Resource 1. Based on the answers given to the questions on age, life expectancy, and current health state, subsequent scenarios were presented graphically and tailored to each respondent’s characteristics (for an example, see Fig. 1). To encounter the problem of high drop-out rates of comparable studies (e.g., 48% [3]), respondents were randomly presented with either the depression or the heart disease questionnaire; and the scenarios within each questionnaire were presented in a random order.

The respondents were presented with a scenario of one health problem (i.e., heart attack or depression) that would hypothetically reduce the respondent’s current health state by a certain amount of health points for a certain amount of time. The respondents were then asked if they would be willing to pay a one-time payment to avoid health loss. If the respondents answered that they *would* be willing to pay money for treatment, a table with three columns was presented, with a series of values in Euros ranging from €10 to €300,000 in accordance with previous studies [3, 4, 21]. To facilitate decision-making, the respondents were asked to sort the Euro values into one of three columns, indicating which amounts they would be willing to pay, would not be willing to pay, and the amounts about which they were unsure. Summarizing the maximum amount, the respondent was willing to pay and the minimum that he or she was not willing to pay, the respondent was asked to state his or her maximum WTP in an open-ended response. If the respondent answered that she or he was *not* willing to pay money to avoid health loss, he or she was asked to indicate one of numerous reasons from a set of pre-coded responses, or by using a free text option. These statements were directly translated from the EuroVaQ questionnaire [3, 21]. The remaining three scenarios were presented in a similar manner. The sequence and translation of one exemplary scenario is presented in Online Resource 2. Lastly, respondents were asked to rate how much they currently knew about the treatment method of electroconvulsive therapy (in the depression questionnaire) or bypass operation (in the heart disease questionnaire) and were asked to state whether they thought this method was adequate. Respondents were given the chance to view all their answers on one page, and to validate or change their answers.

### Health gains valued

Respondents were presented with health gains of either one QALY (scenarios A and B) or a fraction of a QALY (scenarios C and D). All scenarios are in the style of the EuroVaQ scenarios. An overview of the four scenarios can be found in Table 1.

**Table 1:** Health gains valued for the two questionnaire versions

Scenario description	Health loss	Duration	Point in time	Health gain through treatment (%)	Treatment
Questionnaire version: depression					
Scenario A	25 points	4 years	In 1 year	100	Pain-free treatment
Scenario B	10 points	10 years	In 1 year	100	Pain-free treatment
Scenario C	25 points	4 years	In 1 year	90	Inpatient treatment (8 weeks)
Scenario D	25 points	4 years	In 1 year	90	Inpatient treatment (8 weeks) plus electroconvulsive therapy
Questionnaire version: heart disease					
Scenario A	25 points	4 years	In 1 year	100	Pain-free treatment
Scenario B	10 points	10 years	In 1 year	100	Pain-free treatment
Scenario C	25 points	4 years	In 1 year	90	Inpatient treatment (8 weeks)
Scenario D	25 points	4 years	In 1 year	90	Inpatient treatment (8 weeks) plus bypass operation

To test for differences across scenarios, we investigated the following hypothesis (holding the illness-specific context constant):

#### Hypothesis 3

Mean WTP is significantly higher in scenario A compared to scenario B in both questionnaire versions.

#### Hypothesis 4

Mean WTP is significantly higher in scenario D compared to scenario C in both questionnaire versions.

### Recruitment of subjects

Respondents were recruited from an Internet panel run by *USUMA GmbH* (http://www.usuma.com). The survey was launched on March 6, 2019 and closed on March 25, 2019. To achieve representativeness of the German general public by age, gender, socioeconomic status and region both within and across the total sample, respondents were allocated to one of the two questionnaire versions randomly until quotas for socio-demographic characteristics were achieved.

### Exclusion criteria

**Fig. 2:**
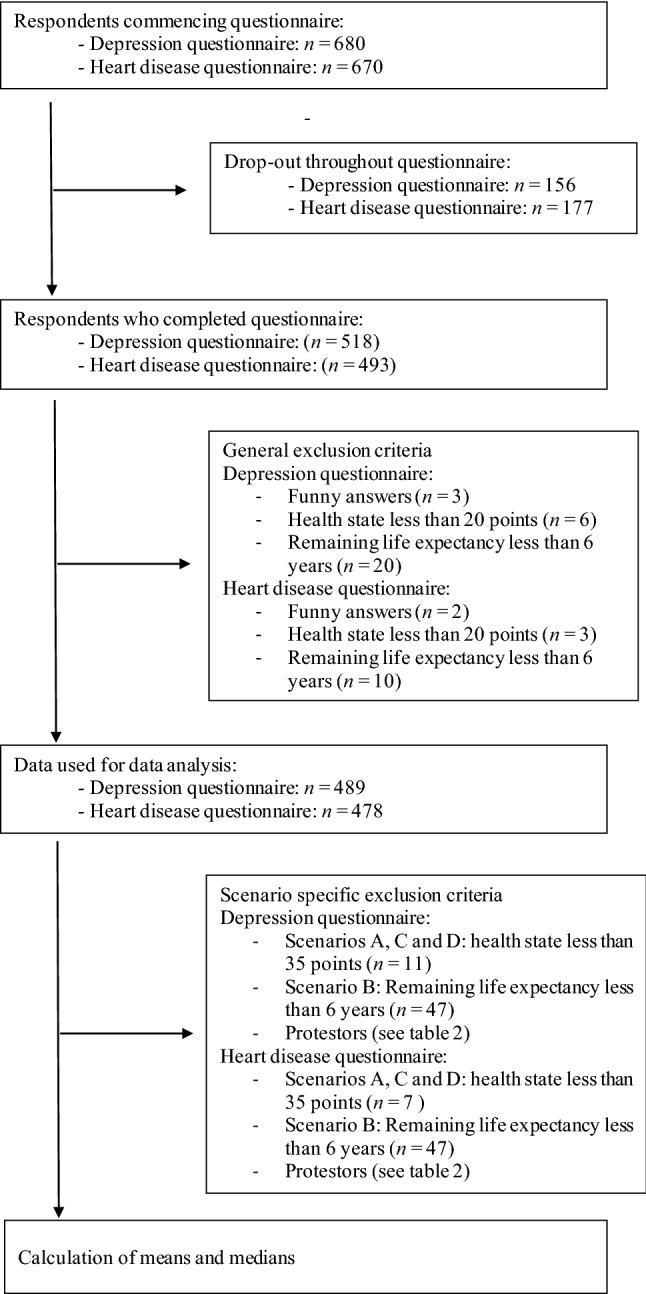
Flowchart

To ensure that the questions were relevant to the individual respondents, and to ensure comparability to the EuroVaQ report, the following exclusion criteria were applied: Respondents were excluded from all data analysis if (a) their health state was less than 20 points, and (b) their life expectancy was less than 6 years. Additionally, respondents were excluded from data analysis regarding scenarios A, C, and D if (c) they rated their health state at less than 35 points, and excluded from data analysis regarding scenario B if (d) life expectancy was assumed to be below 12 years. The intention was to ensure that no health loss reduced the respondent’s health to below 10 points and that all health gains were complete at least 1 year before the respondent expected to die. A total of five respondents were excluded based on the following reasons: respondents indicating an implausibly high number of people living in the household (*n* = 3) and an implausible age (*n* = 2). The flowchart in Fig. 2 shows the process of data analysis. The primary analysis reported here has been undertaken on the set of complete answers. As is conventional in WTP studies, ‘protest respondents’ who were not willing to pay to avoid health loss for the sole reason that “the government should pay” were excluded, because the respondents did not seem to understand the hypothetical nature of the question (see [3, 4, 21, 22]).

### Data analysis

All analyses was undertaken in IBM SPSS Statistics 26. Using open-ended questions allowed determination of the mean and median values reported for each scenario. WTP values were collected in 2019 Euros and are also reported in US dollars to facilitate international comparability. In accordance with the standard procedure in WTP studies, we report trimmed means and medians (excluding the top 1% of WTP responses) to reduce the impact of extreme, possibly implausible upper-end responses.

The Kolmogorov–Smirnoff test was used to test the assumption of normal distribution. WTP scores for scenario A (*D*(629) = 0.450, *p* < 0.001), scenario B (*D*(570) = 0.393, *p* < 0.001), scenario C (*D*(563) = 0.432, *p* < 0.001), and scenario D (*D*(514) = 0.430, *p* < 0.001) all differed significantly from normal. Because of unmet normality assumption, bias-corrected and accelerated 95% confidence intervals around means were estimated using a two-stage bootstrapping routine [23, 24]. To reduce the impact of outliers on results, and because of the skewed distribution, the nonparametric Mann–Whitney *U*-test and Median test were used to assess whether responses differed by questionnaire version (i.e., depression questionnaire vs. heart disease questionnaire) on WTP per QALY. Effect size *r* was calculated [24, 25]. To test hypothesis 2—whether the likelihood of expressing a positive WTP differed across questionnaire versions—WTP responses were dichotomized as zero and non-zero values, and Pearson’s chi-square tests and odds ratios were calculated for all scenarios. To analyze whether mean WTP responses differed significantly across scenarios (holding the illness-specific context constant), paired *t*-tests for the respective question pairs were conducted. A Bonferroni corrected *α* (*P*_crit_ = *α*/*k* = 0.05/2 = .025) was applied. A logistic regression was used to investigate the effects of respondent’s characteristics (age, sex, income, educational level, current health state and PHQ-2 score) on the likelihood of indicating positive WTP.

## Results

A total of 967 respondents answered each of the four questions across the two questionnaire versions. Dropout rates were 23% for the depression questionnaire and 26% for the heart disease questionnaire and were comparable to dropout rates for other surveys from this company. Most respondents (9.7%) dropped out immediately after reading about the objective of the study and before giving consent. No effect of gender, age or health status was found on drop-out. Respondents’ characteristics are displayed in Table 2. The final sample was broadly representative of the German general public. The mean age of the respondents was 48.52 years, and 49.94% were male. No between-group differences were found in terms of sociodemographic data and the frequencies of pre-existing conditions (i.e., mental disorders, heart disease). Table 3 reports number of respondents, number of zero WTP, and number of protestors by questionnaire version.

**Table 2:** Respondents’ characteristics

Characteristic	Questionnaire: heart disease *N* = 478		Questionnaire: depression *N* = 489	
	*M* (SD)	Min/max	*M *(SD)	Min/max
Age (in years)	48.68 (16.89)	18/89	48.35 (17.09)	18/83
Size of household	2.5 (1.24)	1/9	2.27 (1.11)	1/6
Number of children	1.02 (1.16)	0/7	.95 (1.11)	0/6
Life expectancy^a^ (age)	83.11 (9.94)	30/150	83.39 (10.76)	40/150
Own health (20–100)	79.87 (16.44)	20/100	80.24 (16.78)	20/100

	*n*	%	*n*	%
20 to 69 (poor)	88	18.4	85	17.4
70 to 79 (rather poor)	69	14.4	78	16.0
80 to 89 (rather good)	127	26.6	109	22.2
90 to 100 (very good)	194	40.6	217	44.4
Low remaining lifetime (less than 16 years)	89	18.1	88	18.0
Males (rather than females)	230	48.1	253	51.7
Educational level				
Low (up to 10 years of schooling)	71	14.8	83	17.0
Medium (10 years of schooling)	165	34.5	155	31.7
High (additional 3 years of advanced education)	233	48.7	245	50.1
Family status				
Single	158	33.1	170	34.8
Married	228	47.7	229	46.8
Divorced	49	10.3	51	10.4
Widowed	30	6.3	13	.03
Income (monthly net household income €)				
No answer	23	4.8	22	4.5
Below 500 €	8	1.7	11	2.2
500 to below 1.000 €	42	8.8	37	7.6
1.000 € to below 1.500€	51	10.7	55	11.2
1.500€ to below 2.000€	68	14.2	68	13.9
2.000€ to below 3.000€	134	28.0	127	26.0
3.000€ to below 4.000€	87	18.2	96	19.6
4.000€ and more	64	13.4	73	14.9
Health insurance				
No answer	1	.2	2	.4
Public insurance	421	88.1	430	87.9
Private insurance	56	11.7	56	11.5
Pre-existing conditions				
Heart disease (yes rather than no)	45	9.4	46	9.4
Mental disorder (yes rather than no)	96	20.1	104	21.3

*M* mean, *SD* standard deviation, *Min/Max* minimum/maximum, *N* sample size

^a^Excluding respondents with a life expectancy of less than 6 years

**Table 3:** Number of respondents, zero WTP and protestors

Scenario description	*n*^a^	*n* zero WTP (%)	*n* protestors (%)
Questionnaire version: depression			
Scenario A	478	190 (39.7)	32 (6.7)
Scenario B	442	215 (48.6)	29 (6.6)
Scenario C	478	240 (50.2)	43 (9.0)
Scenario D	478	270 (56.5)	39 (8.2)
Questionnaire version: heart disease			
Scenario A	472	145 (30.7)	25 (5.3)
Scenario B	432	147 (34.0)	30 (6.9)
Scenario C	472	157 (33.3)	25 (5.3)
Scenario D	472	177 (37.5)	34 (7.2)

^a^Excluding respondents who met exclusion criteria (a) to (d) but including protestors

Trimmed median, mean, and maximum WTP values and 95% confidence intervals around trimmed means are reported in Table 4. In the depression questionnaire, median WTP values ranged from €1000 to €1500. In the heart disease questionnaire, median WTP ranged from €1000 to €2000. Untrimmed WTP values are reported in Online Resource 3.

**Table 4:** **1% trimmed mean, median and maximum values by scenario and questionnaire version excluding ‘protest respondents’ in Euros

Scenario description	*N*	*n* zero WTP (%)	1% trimmed means	95% CI around trimmed means	1% trimmed median	1% trimmed maximum
Questionnaire version: depression						
Scenario A	443	158 (35.7)	3451 (4170)	2665–4348 (3220–5254)	1000 (1209)	85,000 (102,706)
Scenario B	410	187 (45.6)	2892 (3494)	2172–3730 (2624–4507)	1000 (1209)	50,000 (60,415)
Scenario C	432	197 (45.6)	3610 (4362)	2892–4461 (3494–5391)	1500 (1814)	50,000 (60,415)
Scenario D	436	231 (53.0)	3011 (3638)	2215–3938 (2677–4759)	1000 (1209)	60,000 (72,504)
Questionnaire version: heart disease						
Scenario A	444	120 (27.3)	7676 (9283)	5434–10,358 (6,569–12,522)	1500 (1814)	300,000 (362,670)
Scenario B	399	117 (29.3)	5766 (6971)	4222–7591 (5104–9177)	1000 (1209)	150,000 (181,335)
Scenario C	444	132 (29.7)	5832 (7050)	4624–7137 (5590–8628)	1500 (1814)	150,000 (181,335)
Scenario D	435	143 (32.9)	6844 (8274)	5567–8316 (6730–10,053)	2000 (2418)	150,000 (181,335)

WTP values in U.S. $ are in parentheses. Values in € were converted to U.S. $ at the rate of 1 € = $1.2084 on December 10th, 2020

*n* sample size. *CI* confidence interval

Results from the Mann–Whitney U-Tests and Median test indicate significant differences in distribution across questionnaire versions in scenario B (*U* = 29,065, *z* =  − 2.259, *p* = 0.024, *r* =  − 0.099) and scenario D (*U* = 26,213, *z* =  − 3.064, *p* = 0.002, *r* =  − 0.136). In scenario C, both the Mann–Whitney U-Test and Median test indicated that WTP per QALY regarding heart disease (*Mdn* = €1500) did not significantly differ from the equivalent depression scenario (*Mdn* = €15,000; *U* = 36,262, *z* =  − 0.905, *p* = 0.366, *r* =  − 0.038). For scenario A, the Mann–Whitney *U*-test indicated significant differences (*U* = 43,107, *z* =  − 2.399, *p* = 0.016, *r* =  − 0.096), whereas the Median test showed no significant differences between the depression questionnaire (*Mdn* = €1000) and the heart disease questionnaire (*Mdn* = €1500; *p* = 0.051).

Examining hypothesis 2, Pearson’s chi-square test indicated that there were significant associations between questionnaire version and zero WTP in all scenarios (Scenario A: (*χ*^2^(1) = 7.66, *p* = 0.006); scenario B: (*χ*^2^(1) = 22.14, *p* < 0.001); scenario C (*χ*^2^(1) = 23.41, *p* < 0.001); and scenario D (*χ*^2^(1) = 35.75, *p* < 0.001). Reasons stated for zero WTP are tabulated in Table 5. Odds ratios presented in Table 6 show that the odds of indicating zero WTP were 1.5 times (scenario A) to 2.3 times (scenario D) higher in the depression scenarios compared to the corresponding heart disease scenarios.

**Table 5:** Frequencies of reasons for zero WTP

Scenario	*N* zero WTP	It wouldn’t be so bad/I could live with it	Effects of treatment are too small	I want my family to have the money instead	I would get better without treatment	I value the treatment but can’t afford it	I value treatment but government should pay	Other reasons
Questionnaire version: depression								
A	190 (39.7)	43 (9.0)	25 (5.2)	18 (3.7)	30 (6.3)	31 (6.5)	32 (6.7)	8 (1.7)
B	215 (48.6)	61 (13.8)	39 (8.8)	13 (2.9)	36 (8.1)	36 (7.4)	29 (6.6)	5 (1.1)
C	240 (50.2)	48 (9.8)	42 (8.8)	19 (4.0)	35 (7.3)	34 (7.1)	43 (9.0)	16 (3.4)
D	270 (56.5)	47 (9.8)	51 (10.7)	15 (3.1)	45 (9.4)	30 (6.3)	39 (8.2)	41 (8.6)
Questionnaire version: heart disease								
A	145 (30.7)	21 (4.4)	15 (3.2)	21 (4.4)	13 (2.8)	36 (7.6)	25 (5.3)	14 (2.9)
B	147 (34.0)	25 (5.8)	23 (5.3)	12 (2.8)	24 (5.6)	26 (6.0)	30 (6.9)	7 (1.6)
C	157 (33.3)	29 (6.1)	22 (4.7)	13 (2.8)	24 (5.1)	33 (7.0)	25 (5.3)	11 (2.3)
D	177 (37.5)	22 (4.7)	26 (5.5)	15 (3.2)	24 (5.1)	39 (8.3)	34 (7.2)	17 (3.6)

Percentages are in parentheses

*N* sample size

**Table 6:** Results of Pearson’s chi-square test and odds ratios for zero WTP for depression questionnaire

Scenario	*χ*^*2*^	OR
A	7.66**	1.49
B	22.14**	1.99
C	23.41**	1.98
D	35.75**	2.29

*OR* odds ratio

^**^p < .01

Results of the paired *t*-tests are presented in Table 7. Presenting respondents with the heart disease scenario, respondents were willing to pay significantly more money for an 8-week inpatient treatment including bypass operation (scenario D) compared to an 8-week inpatient treatment only (scenario C). No significant differences were found between scenarios A and B for both questionnaire versions and between scenarios C and D in the depression questionnaire.

**Table 7:** Mean within-respondent differences in values between question pairs (holding illness-specific context constant)

Scenarios compared	*n*^a^	Mean difference	95% CI	*p*-value for paired *t-*test
Questionnaire version: depression				
Scenario A–scenario B	221	1438	− 25 to 2901	.054
Scenario C–scenario D	181	357	35 to 679	.030
Questionnaire version: heart disease				
Scenario A–scenario B	278	9604	− 4357 to 23,566	.177
Scenario C–scenario D	269	− 841	− 1457 to − 225	.008*

*n* sample size, *CI* confidence interval

^a^General exclusion criteria were applied

*p < .025 (Bonferroni corrected α value)

Assessing the effects of respondents’ characteristics on the likelihood of indicating a positive WTP, only income was found to have a significant effect on respondents’ WTP in the depression questionnaire, whereas in the heart disease questionnaire, a male gender and a higher educational level were associated with a higher likelihood of expressing a positive WTP.

## Discussion

To the author’s knowledge, the present study is the first one that compares the value a population-representative sample places on mental versus physical health, in this case, relief from depression or relief from heart disease. Health-gain scenarios customized to fit respondents’ characteristics allowed us to present hypothetical scenarios in a personally relevant matter to a large population representative sample. In the depression questionnaire, median values ranged from €1000 to €1500; in the heart disease questionnaire from €1000 to €2000. Median differences between questionnaire versions were significant in scenarios B and D: It seems that respondents valued QALY gains for cardiovascular health higher compared to QALY gains for depression, when QALY gains were minor and stretched over a long period of time and when treatment with bypass operation (rather than treatment with ECT) was offered. Additionally, results regarding hypothesis 4 indicate that respondents were willing to pay significantly more money for an additional bypass operation in the heart disease questionnaire (scenario D) compared to an eight-week inpatient treatment alone (scenario C). However, no significant differences were found for the equivalent scenarios in the depression questionnaire. Respondents indicated a WTP approximately twice as high for an 8-week inpatient treatment with a bypass operation for heart disease compared to the equivalent depression scenario with ECT. Although the effectiveness of ECT is recognized by the American Psychiatric Association and similar organizations in Germany [26], the rate of ECT use is particularly low in Germany (3.5 per 100,000 inhabitants, compared to 41 per 100,000 inhabitants in Sweden and Belgium [27–29]) and ECT is still offered in less than 50% of Germany’s psychiatric clinics [30]. Interestingly, comparing scenarios A from the assessed health gain scenarios to the equivalent, but generally presented scenario of the EuroVaQ study [3], median WTP values were lower in the depression questionnaire ($1176, compared to $1532 in scenario A of the EuroVaQ study), but comparably higher in the heart disease questionnaire ($1763, compared to $1532).

The number of respondents indicating zero WTP was significantly higher in all scenarios of the depression questionnaire in comparison to the heart disease questionnaire and the odds of indicating zero WTP were up to 2.3 times higher in the depression scenarios. According to the stated reasons for zero WTP—in the depression questionnaire: “It wouldn’t be so bad/I could live with it”; in the heart disease questionnaire: “I value the treatment but can’t afford it”—the necessity of treating a somatic disease, i.e., heart disease, seems to be more prevalent than the necessity of treating a mental disorder, i.e., depression. To date, although depression is one of the most dire, and common global health problems, mental disorders are still associated with stigmatization [31]. In a sample of college-aged individuals, less than 25% of individuals who met the criteria for a mental disorder had sought treatment within the past year [32]. Approximately 70% of people who experience a mental disorder do not seek healthcare treatment [33]. Factors contributing to the gap between true and treated prevalence include lack of knowledge about the symptoms and how to seek treatment, as well as fear due to anticipated or real acts of discrimination against those with a diagnosed mental disorder [34]. In conclusion, fear of stigmatization, lack of knowledge about the symptoms of depressive disorders and their impact on quality of life, and ignorance of the treatment options may be relevant factors associated with indicating zero WTP to the treatment of depression.

### Limitations

Studies have shown that WTP valuations are highly sensitive to framing effects (e.g., [35]). We tried to address such framing effects by maximizing comparability to the EuroVaQ questionnaire [3] in terms of order and wording of the scenarios and using the same contingent valuation methods (binary response filter, payment cards and open-ended response format). Within each questionnaire version, all scenarios were randomized to control for order effects. However, it is possible that recruitment method and the exclusion of incomplete answers has led to bias in the estimates. It should also be noted that using an ex-post perspective, as in the present study (i.e., respondents are asked to imagine having experienced a heart attack/depressive symptoms), usually results in lower WTP estimates than valuing WTP from an ex-ante perspective (where WTP is evaluated previous to the existence of the need; e.g., [36, 37]).

Additionally, the hypothetical scenarios in which the success of treatment is certain and will fully restore the respondents’ initial health state—which is highly unlikely—may have led to overestimation of WTP estimates. More realistic health-gain scenarios with uncertainty characteristics should be evaluated in further research. As health insurance is mandatory in the German health care system, we included an introductory statement in accordance with Ahlert and colleagues [4] to emphasize the hypothetical scenario that such a mandatory health insurance does not exist. However, the use and wording of such introductory statements should further be evaluated. Results could also be influenced by respondents’ perception that heart disease is associated with a higher reduction in quality of life and is more lethal than depressive disorders. We tried to maximize comparability of both health states by describing both health scenarios in a similar manner, including annual deaths by suicide for the depression questionnaire and annual deaths by heart attack for the heart disease questionnaire. Nevertheless, annual deaths by heart attack are approximately five times higher than deaths by suicide in Germany, which may have influenced WTP statements. Related studies should include a broader variety of versions of questionnaires, as proposed by Ahlert et al. [38], and should evaluate the wording of health-state descriptions. Additionally, an end-of-life scenario should be included, as it might be especially relevant in the development of governmental healthcare policies and decisions regarding the treatment of cardiovascular diseases.

External validity may be limited, as respondents from the public may not have been able to relate to the presented scenarios as well as patients who experience depressive disorders or heart diseases. Therefore, further research should investigate the effects of respondents’ characteristics on WTP per QALY; specifically, if respondents with pre-existing depressive disorders or heart diseases are placing higher values on QALY gains for the respective health-state scenario. Caution is also needed in interpreting and generalizing the results of the present research. Recent studies indicate that WTP per QALY seems to depend on several differential contextual factors, such as the size of the QALY gain valued, illness type and severity (e.g., [6, 7]), and the design and wording of the questions presented [38]. The present research supports the concluding remarks of recent studies: the assumption that “a QALY is a QALY is a QALY” and the determination of a uniform price threshold per QALY seem less than likely [6, 7]. Therefore, caution is needed when transferring the values placed on QALY gains for heart disease and depression to other mental disorders or physical illnesses. In addition, we derive WTP per QALY based on individual preferences. From a societal perspective, and if WTP per QALY estimates are used for health care decisions, indirect health care costs (such as productivity loss and sick leave) should also be taken into consideration, possibly resulting in higher values per QALY gain [39].

## Conclusions

Having presented respondents with different hypothetical QALY gains for a mental or physical health scenario (depression and heart disease), this study supports previous findings that the determination of a uniform price threshold for a QALY gain may not be suitable. Although recent studies have shown that WTP is not linearly proportional to the QALY gains valued, and that it may not be empirically attainable to estimate a single QALY value (e.g., [6, 7]), estimating a population’s WTP per QALY may still be an important input for policy- and decision-makers, as it facilitates an understanding of the population’s preferences regarding resource allocation. As concluded by Sund and Svensson [1], a specified threshold value per QALY based on state-of-the-art research may improve efficiency when deciding which treatment interventions to fund or reimburse.^2^

## Supplementary Information

Below is the link to the electronic supplementary material.

Supplementary Information 1 (DOCX 50 KB)

## Data Availability

The data that support the findings of this study are available on request from the corresponding author. The data are not publicly available due to privacy or ethical restrictions.

## Footnotes

1 Germany has a universal health care system with two types of health insurance: Germans can choose between public (statutory) or private health insurance, which are co-financed by employer and employee. As of today, no threshold per QALY exists.

2 Such thresholds have been introduced for example in the United Kingdom and the Netherlands. For further information and a discussion of cost-effectiveness thresholds, readers are pointed to [40].
