# Supplementary material for: Willingness to pay for a quality-adjusted life year for depressive disorders compared to heart disease based on population preferences
Source: Qual Life Res. 2021 Feb 15;30(7):1985–95. doi: 10.1007/s11136-021-02772-x (PMC8233267; doi:10.1007/s11136-021-02772-x)
Supplement: Supplementary file 1 — Supplementary Information 1 (DOCX 50 KB) [file 11136_2021_2772_MOESM1_ESM.docx]

**Online Resources**

**Online Resource 1** Translation of the health state descriptions

Cardiovascular disease

“In this survey, we ask for your willingness to pay for a treatment of one of two different diseases (depressive episode and heart attack. In the following abstract, we present the clinical picture of a heart attack.

A heart attack is an acute and life-threatening event which can occur due to a sustained disturbed blood flow of parts of the heart muscle. The primary symptom of a heart attack is a sudden, prolonged, and usually intense pain in the chest, that radiates predominantly in the left side to shoulders, arms, lower jar, back and upper abdomen. It is often accompanied by cold sweat, nausea, and possibly vomiting. According to the Federal Statistical Office, in 2015 alone, more than 50.948 individuals in Germany died due to a heart attack. After having experienced a heart attack, most affected persons live in fear of experiencing another heart attack. Within 6 months, five percent of all affected persons experience another heart attack, which doubles the risk of death, then estimated at 60 percent. The heart’s pumping power is reduced, so that most affected persons suffer from shortage of breath and loss of efficiency.

As mentioned above, we would like to investigate your willingness to pay money for treatment of a heart attack. We would therefore like to ask you to imagine that there are no insurance companies in Germany and that you would not have to pay contributions or premiums for health insurance. Your net income would therefore be higher by that respective amount. Instead, you would have to pay for medical treatments out of your own pocket. As you have known this for a long time, you have savings in the amount of a yearly income to be prepared for unexpected medical costs.

The amount of money you are willing to pay for a treatment provides an indication of the value you attribute to the increase of your well-being and your health achieved by this treatment method (compared to other treatment options).”

Depressive disorder

“In this survey, we ask for your willingness to pay for a treatment of one of two different diseases (depressive episode and heart attack. In the following abstract, we present the clinical picture of a depressive episode.

A depressive episode is a mental disorder. Common symptoms are a depressed mood, rumination and feeling unpowered. Often, joy and the feeling of delight, productivity, empathy, and the will to live are lost. Those symptoms can be experienced by health people for a short period of time. Within a depressive episode, these symptoms are experienced for a longer period a time, are more severe and significantly reduce quality of life. Other common symptoms are hopelessness and suicidal ideation and impulses. According to the Federal Statistical Office, in 2015 alone, 10.078 people in Germany died due to suicide.

As mentioned above, we would like to investigate your willingness to pay money for treatment of a depressive episode. We would therefore like to ask you to imagine that there are no insurance companies in Germany and that you would not have to pay contributions or premiums for health insurance. Your net income would therefore be higher by that respective amount. Instead, you would have to pay for medical treatments out of your own pocket. As you have known this for a long time, you have savings in the amount of a yearly income to be prepared for unexpected medical costs.

The amount of money you are willing to pay for a treatment provides an indication of the value you attribute to the increase of your well-being and your health achieved by this treatment method (compared to other treatment options).”

**Online Resource 2** Exemplary process of the survey

1. **Presentation of the health state description**

See Online Resource 1 for the translation of the two health state descriptions

1. **Presentation of the scenario: Example of scenario A of the CVD questionnaire**

“Please imagine that in one year - instead of living the next *4 years* at your current health state - you would suffer from a *heart attack*, which would reduce your health state *by 25 points* on the health thermometer. Personally, that would mean that your health would be reduced from *90 points to 65 points* (see figure below).

After these four years, at the *age of 33*, your current health state would be restored for the rest of your life.”


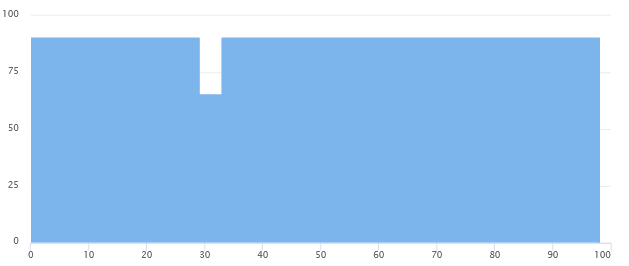


health state

Age (in years)

1. **Asking for willingness to pay:**

“Would you be willing to pay money now (even if it is only a small amount) for a *simple, safe and pain-free treatment*, which would restore your initial health state after experiencing a heart attack?”


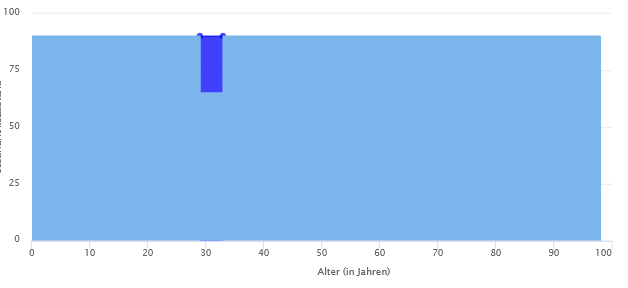


health state

Age (in years)

**4a. If respondent states “No”**

“Can you say why you are not willing to pay money for a treatment, that would prevent a reduction of your health state by *25 points* over a period of *four years*, beginning in one year?”

Please choose the main reason why you are not willing to pay money for treatment:

- “It would not be too bad./ I could live with it.”
- “Effects of treatment are not sufficient.”
- “I am thinking about my family/my partner. I want them to have the money instead.”
- “My health would probably improve without treatment.”
- “I value the treatment but cannot afford it at the moment.”
- “I value the treatment but I don’t want to pay for it. The government should pay for health care costs.”
- Other reasons (please specify).

**4b. If respondent states “Yes”**

“You said that you would be willing to pay money for a treatment that would prevent a reduction of your health state by *25 points* over a period of *four years* at the age of 29.

What would be the highest one-time payment that you would be willing to pay today at the age of 28 for this treatment, imagining that there are no insurance companies that would pay and if you had savings in the amount of one year’s income?”

*Presentation of a table with Euro values that can be sorted into one of three columns: “Willing to pay”, “Not willing to pay” and “Unsure” and open-ended response*

**5. Asking about current knowledge regarding ECT/bypass operation**

“How much do you currently know about treatment of ECT/bypass operation?”

- Nothing
- a little
- some
- much
- very much

“How expedient do you think ECT/bypass operation is?”

- Not at all expedient
- a little expedient
- some
- expedient
- very expedient

**Online Resource 3** Untrimmed mean, median and maximum values by scenario and questionnaire version excluding ‘protest respondents’ in Euros

| Scenario description | *N* | *n* Zero WTP (%) | *M* | 95% CI around means | Median WTP | Maximum WTP |
| --- | --- | --- | --- | --- | --- | --- |
| Questionnaire version: Depression | | | | | | |
| Scenario A | 446 | 158 (35.4) | 5,325  (6,260) | 3,294-7,888  (3,872-9,272) | 1,000  (1,176) | 300,000  (352,650) |
| Scenario B | 413 | 187 (45.3) | 3,606  (4,239) | 2,486-5,017  (2,922-5,897) | 1,000  (1,176) | 70,000  (82,285) |
| Scenario C | 435 | 197 (45.3) | 5,561  (6,537) | 3,512-8,408  (4,128-9,884) | 1,500  (1,763) | 300,000  (352,650) |
| Scenario D | 439 | 231 (52.6) | 5,251  (6,173) | 3,052-8,495  (3,588-9,986) | 1,000  (1,176) | 300,000  (332,545) |
| Questionnaire version: Heart disease | | | | | | |
| Scenario A | 447 | 120 (26.8) | 15,581  (18,331) | 7,651-28,778  (8,995-33,831) | 1,500  (1,763) | 2,000,000  (2,351,000) |
| Scenario B | 402 | 117 (29.1) | 7,986  (9,396) | 5,081-11,605  (5,973-13,643) | 1,000  (1,176) | 300,000  (352,650) |
| Scenario C | 447 | 132 (29.5) | 9,980  (11,741) | 5,587-15,499  (6,558-18,221) | 1,500  (1,763) | 1,000,000  (1,175,500) |
| Scenario D | 438 | 143 (32.6) | 11,282  (13,273) | 6,414- 18,750  (7,540-22,043) | 2,000  (2,351) | 1,000,000  (1,175,500) |

*Note.* WTP values in U.S. $ are in parentheses. Values in EUR were converted to U.S. $ at the rate of 1 EUR = $1.1756 on September 22^nd^, 2020. *n* = sample size; *M* = mean; CI = confidence interval.
